# An Overview of the Impact of Melatonin on Tomato's Abiotic Stress Responses

**DOI:** 10.1155/sci5/8896081

**Published:** 2025-11-06

**Authors:** Ika Cartika, Rinda Kirana, Rahmat Budiarto, Syariful Mubarok

**Affiliations:** ^1^Faculty of Agriculture, Universitas Padjadjaran, Sumedang, Indonesia; ^2^Research Center for Horticultural, National Research and Innovation Agency, Cibinong, Indonesia; ^3^Department of Agronomy, Faculty of Agriculture, Universitas Padjadjaran, Sumedang, Indonesia

**Keywords:** abiotic stress, drought, heat stress, melatonin, salinity, tomato

## Abstract

Tomatoes, as one of the main vegetables in the world, are facing increasingly greater cultivation challenges due to environmental changes. The challenges include abiotic stresses such as high temperatures, drought, and salinity, which significantly impact tomato growth. Melatonin, a hormone recognized for its function in the human sleep cycle, has surfaced as a potential remedy for improving plant resilience. This article explores the effects of melatonin on tomatoes, particularly under abiotic stress. A comprehensive literature search using Scopus, ScienceDirect, Web of Science, PubMed, and ProQuest ensures the synthesis of relevant scientific literature. This review reports that the melatonin content in tomatoes varies among plant organs and is influenced by environmental factors. The application of exogenous melatonin has been proven beneficial in stress conditions, enhancing antioxidant activities, photosynthesis, and stress resistance. The function of melatonin in gene regulation, antioxidants, and signaling pathways contributes to stress adaptation. The biosynthesis of melatonin in plants includes the enzymatic conversion of tryptophan. Although much is already known, future research is essential to comprehend the function of several melatonin features in sustaining plant equilibrium under environmental stresses.

## 1. Introduction

Tomatoes are one of the main types of vegetables in the world. Tomatoes ranked first among the most produced vegetables in the world with 189 million tons in 2021, followed by onions (107 million tons), cucumbers (93 million tons), and cabbage (71 million tons), and as the main processed vegetable [1]. Tomatoes can be consumed fresh or used as a base for food processing [1, 2]. Tomato sauce, tomato soup, and tomato paste are examples of foods made from tomatoes. Tomatoes are low in calories and contain essential nutrients such as minerals, vitamins, proteins, essential amino acids, fatty acids, and carotenoids that are beneficial for health [3]. The lycopene content in tomatoes can reduce the risk of heart disease and cancer [4]. Tomatoes are not only one of the most popular vegetables in the world but also contribute to the global industry and human health.

Tomatoes are cultivated in both tropical and subtropical climates. Tomato plants require certain environmental conditions to grow optimally. Tomato plants require a soil pH of 6.0–6.8, as well as plenty of light and water. The optimal temperature for vegetative growth and fruit development of tomatoes is 18°C–24°C [5]. The yield of tomato cultivation will be optimal if the environmental conditions support their growth. The challenges faced in tomato cultivation are increasing due to rapid environmental changes. Extreme temperature changes, drought, and salinity are among the most significant limiting factors that significantly affect growth and can reduce tomato production. Extreme temperatures cause oxidative stress, drought disrupts hormonal balance and inhibits the rate of plant photosynthesis, and salinity stress negatively impacts the plant's capacity to absorb water [6]. These conditions necessitate creative ways to improve plant resilience against diverse pressures [7]. One interesting approach is the use of N-acetyl-5-methoxytryptamine (melatonin), a hormone long known in relation to the human sleep cycle [8], but now also becoming a significant focus of research in the field of agriculture [9]. Despite increasingly difficult environmental challenges, it is hoped that melatonin will become a solution to enhance tomato production.

Melatonin is a significant bioactive molecule synthesized from tryptophan in vascular plants, identified in 1995 [10]. Initially, melatonin was considered an antioxidant with different roles in various stages of plant growth and development [11], such as germination [12], root elongation [13], leaf senescence [14], and photosynthesis [15]. Melatonin is a phytochemical that significantly contributes to the promotion of plant development and regulation [13]. Melatonin has been identified in diverse plant tissues, including seeds, roots, leaves, and fruits [12]. The potential role of melatonin in enhancing plant growth and regulation has begun to be investigated by researchers [16–18]. Melatonin functions as a biostimulant for plant growth and development [19, 20], plays a role in responding to various environmental stresses [21–23], can enhance growth, reduce oxidative stress [24, 25], and improve harvest quality [26]. Seeing the many benefits of melatonin related to its effects on plant growth, it is worth further research in the field of agriculture.

Melatonin has emerged as an interesting candidate in efforts to enhance plant resistance to abiotic stress [19, 27–30]. Nonetheless, some facets about the mechanism of action of melatonin in tomato plants and its impact on abiotic stress require additional elucidation. This article examines the literature about the impact of melatonin on tomato plants subjected to abiotic environmental stresses, including drought, temperature, and salinity. We will explore the mechanism of action of melatonin in enhancing plant resilience, as well as provide insights into its potential practical applications in agriculture, particularly in tomato plants.

## 2. Materials and Methods

This study employed a systematic literature review (SLR) approach based on the Preferred Reporting Items for Systematic Reviews and Meta-Analyses (PRISMA) [31, 32] guidelines to identify, screen, and analyze relevant literature on the topic of “melatonin, plants, tomatoes, and abiotic stress” from various databases. Data were gathered from five major sources: Scopus, ScienceDirect, Web of Science, PubMed, and ProQuest. Literature searches were conducted using specific keyword combinations, such as “melatonin,” “melatonin and plant,” “melatonin and plant and tomato,” and “melatonin and plant and tomato and abiotic stress,” as shown in Table 1, yielding an initial total of 3861 articles.

**Figure 1:**
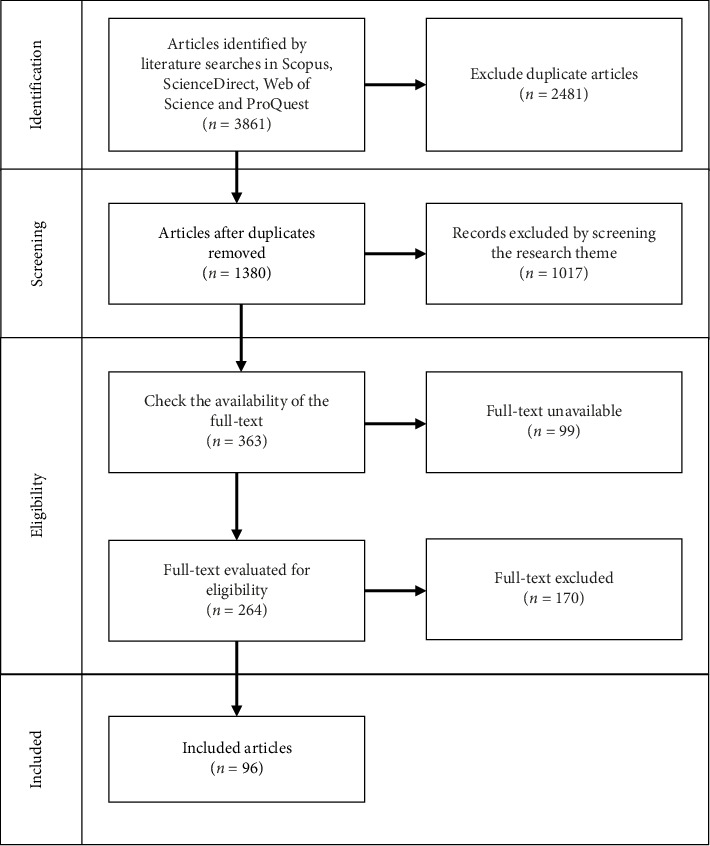
PRISMA flow diagram.

The SLR process followed the PRISMA flow, comprising four main stages: identification, screening, eligibility, and inclusion (Figure 1). In the identification stage, articles from all five databases were combined to gather relevant literature based on the specified keywords. This initial search returned 3861 articles, with 2481 duplicates removed. In the screening stage, the remaining 1380 articles were further assessed for relevance to the research theme, resulting in the exclusion of 1017 articles that did not align with the study's focus. In the eligibility stage, the availability of the full text was checked for the 363 remaining articles. Ninety-nine articles were excluded due to the unavailability of full text, leaving 264 articles for further evaluation. Finally, in the inclusion stage, these 264 articles were assessed against stricter inclusion criteria, resulting in the exclusion of 170 articles. A total of 96 articles were thus selected for detailed analysis.

## 3. Endogenous Melatonin in Tomatoes

Melatonin was first discovered in animals but has now been found in many plants [33]. The concentration of melatonin in plants varies not only from one species to another but also among varieties within the same species. The differences in melatonin concentration in tomato plants among varieties are shown in Table 2. The melatonin content in tomato fruits among varieties varies between 0.64 and 50.1 ng/g. This not only reveals the allocation of melatonin in various tomato plant varieties but also indicates the importance of melatonin in supporting the growth and development of tomato plants.

Melatonin is found in various organs of tomato plant species such as leaves, stems, roots, fruits, and seeds [38]. Initially, melatonin was detected in vegetables and fruits with values ranging from 0 to 862 pg/mg [10]. The melatonin concentration differs among specific plant organs or tissues. Based on Table 3, the melatonin content in the leaves, stems, roots, flowers, fruits, and seeds of tomatoes ranges from 0.03 to 142.5 ng/g. The leaves have the highest melatonin content, and the content in the seeds is higher than in the fruits and flowers. These findings demonstrate the potential of melatonin in enhancing the quality and productivity of tomato plants, as well as its importance in many facets of plant physiology.

Environmental conditions affect the melatonin content of various plant organs. The melatonin levels in the roots, stems, and leaves of tomatoes fluctuate depending on the light–dark period of the plants [36]. Tomato plants under stressed environmental conditions produce more melatonin compared to plants under controlled conditions [39]. Melatonin affects the developmental phases of plants [17], and there is an indication that melatonin concentration increases during the maturation phase [38]. Overall, melatonin has a significant influence on adaptation to changing environments and demonstrates the complexity of melatonin regulation in tomato responses to environmental stresses. This discovery can establish a basis for formulating more effective ways to improve plant resilience to diverse environmental circumstances.

## 4. Biosynthesis of Melatonin in Plants

**Figure 2:**
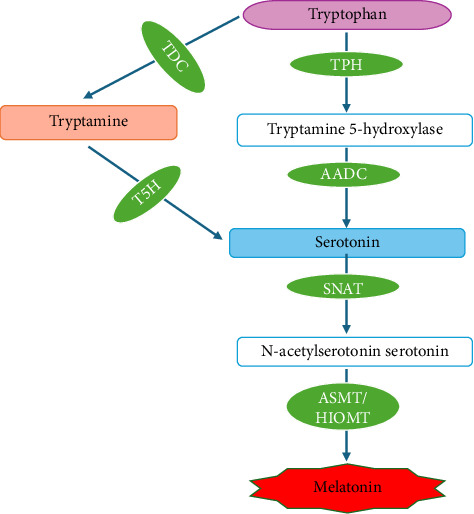
Biosynthesis pathway of melatonin in plants. Tryptophan hydroxylase (TPH), tryptophan decarboxylase (TDC), tryptamine 5-hydroxylase (T5H), aromatic L-amino acid decarboxylase (AADC), serotonin N-acetyltransferase (SNAT), acetylserotonin O-methyltransferase (ASMT), and hydroxyindole O-methyltransferase (HIOMT).

The biosynthesis of melatonin in plants is a multistep process that begins with the essential amino acid tryptophan, which is also the basic material for auxin biosynthesis. Chloroplasts and mitochondria are the main sites of melatonin biosynthesis [42]. The biosynthesis pathway of melatonin is presented in Figure 2. The first step is the hydroxylation of tryptophan catalyzed by the enzyme tryptophan hydroxylase (TPH), converting it into 5-hydroxytryptophan. This hydroxylation process distinguishes melatonin synthesis in animals, where tryptophan undergoes decarboxylation first and is converted into tryptamine, catalyzed by the enzyme tryptophan decarboxylase (TDC) [19]. The subsequent biosynthesis of melatonin involves the enzyme aromatic L-amino acid decarboxylase (AADC), which plays a role in the decarboxylation of 5-hydroxytryptophan, converting it into serotonin. Next, serotonin is converted into N-acetylserotonin by the enzyme serotonin N-acetyltransferase (SNAT). The final stage of melatonin biosynthesis is the methylation of N-acetylserotonin into melatonin. The enzyme acetylserotonin O-methyltransferase (ASMT) or hydroxyindole O-methyltransferase (HIOMT) methylates the hydroxyl group, producing melatonin [23, 43].

At the cellular level, melatonin is produced and actively participates in various important organelles that function in metabolism and protection against oxidative stress. Chloroplasts are one of the main sites of melatonin production due to their role in photosynthesis, which generates free radicals [44]. Melatonin protects chloroplasts from oxidative damage and enhances photosynthetic efficiency. In the mitochondria, which are the centers of cellular energy production, melatonin aids in safeguarding cells from oxidative stress induced during respiration and maintains the function of mitochondria in producing adenosine triphosphate (ATP) [45].

In addition, peroxisomes, which play a role in detoxification and regulate the production of hydrogen peroxide (H_2_O_2_), are also important sites for melatonin activity. Melatonin works to neutralize H_2_O_2_ and protect peroxisomes from oxidative damage [46]. The cytoplasm is also an important location where melatonin acts as a free antioxidant, helping to maintain the oxidative balance of the cell and protect cellular components from damage. Inside the cell nucleus, melatonin helps protect DNA from oxidative damage, which is important in maintaining genetic stability, especially during environmental stresses. Finally, in the endoplasmic reticulum (ER), melatonin helps maintain the balance of proteins and lipids produced there, protecting protein synthesis functions and maintaining cellular homeostasis [47]. With its presence in various organelles, melatonin plays an important role in cellular defense and plant adaptation to environmental stresses.

## 5. Abiotic Stress Affects Plant Melatonin Content

Multiple variables can enhance the manufacture of melatonin in plants. Light is a factor that influences melatonin production [48]. The flowering process, fruit ripening [49], leaf development [38], and leaf senescence [50] also determine the melatonin content in plant organs. Environmental stressors, including ultraviolet-B (UV-B) [51], drought [52], and extreme temperatures [53], are also involved in stimulating melatonin biosynthesis. These factors play an important role in determining the melatonin levels that help plants adapt when environmental changes occur.

The concentration of melatonin within the cells is highly responsive to external conditions. Rapid changes in light, temperature, and various environmental stresses can increase melatonin levels [54]. The melatonin levels of plants vary in different environments, especially under stress conditions [55]. Drought and salinity affect the melatonin content in plants [37, 56]. In addition, the concentration of melatonin in plants is influenced by temperature [57]. Another study states that the melatonin content in plants will increase in response to heat stress, reducing plant tissue damage due to heat stress [36]. A study evaluating the effects of melatonin on tomato seedlings under high-temperature stress showed that melatonin treatment could increase endogenous melatonin levels and photosynthetic pigment content [58]. This suggests the possible application of melatonin as a growth enhancer or plant defense agent in agriculture, especially under extreme environmental conditions.

Environmental factors significantly influence the melatonin levels in plant tissues. Plants cultivated indoors under regulated environmental circumstances exhibit reduced melatonin levels compared to those grown in the field with more diverse environmental conditions. Plants cultivated in direct sunshine possess three times more melatonin in their roots and 2.5 times more in their leaves than those grown under artificial light [59]. Tomato fruits grown in the shade have lower melatonin content compared to those grown without shade [35]. Exposure to UV-B irradiation can elevate melatonin levels in plant roots. Rice seedlings at high temperatures and in dark conditions show increased melatonin synthesis due to enhanced activity of SNAT and ASMT [60]. In addition, plants show varying susceptibility to ozone damage with different melatonin content. Plant species exhibiting greater resistance to ozone damage possess elevated melatonin levels relative to more susceptible species [10]. Melatonin synthesis escalates in response to stress, believed to be an adaptive mechanism for enduring adverse environmental situations. Consequently, melatonin's primary function may be associated with photosynthesis or photoprotection activities.

## 6. The Effect of Exogenous Melatonin on Tomatoes Under Abiotic Stress Conditions

Melatonin shares the same precursor molecule as auxin, namely tryptophan, suggesting that melatonin may also influence plant growth and development similarly to auxin.  Table 4 encapsulates the research findings about the effects of exogenous melatonin on tomato plants, namely, its influence on their morphology, physiology, and quality under conditions of drought, severe temperature, and salinity. The findings of this study demonstrate that exogenous melatonin improves antioxidant activities, promotes photosynthetic performance, and confers tolerance to drought, severe temperatures, and salinity stress.

Drought stress poses a significant hazard to the growth, survival, and production of plants. In response to this ambient state, plants initiate diverse physiological, biochemical, and molecular mechanisms to adapt and survive [73]. One of the components in this adaptation process is melatonin, which is one of the plant growth regulators. The results of several studies indicate that melatonin plays an important role in regulating the photosynthesis process and supporting the antioxidant defense system when plants experience drought stress [74–76]. Melatonin has been proven effective in reducing the decline in plant growth rates [77]. In addition, melatonin contributes to the increased efficiency of photosynthesis, protecting chlorophyll during drought [78]. Melatonin is depicted as a stress-protective agent that provides adaptive mechanisms to ensure reproduction and enhance antioxidant defense systems and photosynthetic activity.

Photosynthesis serves as a crucial indicator of temperature stress, as it is frequently suppressed prior to impacting other cellular functions. Melatonin has emerged as a promising strategy to mitigate the adverse effects of temperature stress [65]. A study focusing on tomato photosynthesis under heat stress revealed that melatonin application increases the net photosynthesis rate and chlorophyll fluorescence. Moreover, melatonin serves a protective function in Photosystem II by facilitating balanced electron transfer at the reactive center and acceptor, thus mitigating oxidative stress and damage to the photosynthetic apparatus [79]. Plants treated with melatonin experienced a slowdown in leaf senescence due to heat, as evidenced by a decrease in yellowing leaves, an increase in the Fv/Fm ratio, and a reduction in reactive oxygen species (ROS) formation. This effect results from melatonin's role in downregulating the expression of essential genes associated with ROS generation (e.g., RBOHS), thereby reducing the rate of chlorophyll degradation and aging. In addition, the treatment of exogenous melatonin on tomatoes causes an elevation in endogenous melatonin and gibberellin concentrations while concurrently diminishing abscisic acid (ABA) levels [67]. Consequently, melatonin may improve plant resilience to thermal stress, safeguard photosynthetic processes, and retard leaf senescence.

Exogenous melatonin treatment on tomato plants subjected to heat stress can stimulate the expression of heat shock protein (HSP) genes [57]. HSPs are a group of proteins produced by cells in response to exposure to stress conditions, such as high temperatures. HSPs play a role in protecting cells from damage and helping cells recover from stress effects [80]. HSPs are known to be involved in various cellular processes, including protein folding, assembly, and transport, as well as in regulating the cell cycle and responding to DNA damage [81]. The application of exogenous melatonin to tomato plants under heat stress has been demonstrated to stimulate the production of HSP genes. This indicates that melatonin may assist plants in managing heat stress by promoting the synthesis of HSPs, which in turn can help protect plants from heat damage.

Salinity stress presents considerable obstacles to plant productivity and profoundly affects physiological and biochemical processes, including photosynthesis. Numerous studies indicate that melatonin significantly mitigates stress effects by enhancing the levels of photosynthetic pigments and bolstering antioxidant defenses. Tomato plants subjected to natrium chloride (NaCl) stress, upon administration of exogenous melatonin, exhibit reduced chlorophyll breakdown, modulation of photosynthetic electron flow to mitigate ROS production, and enhanced activity of enzymes associated with the ascorbate–glutathione cycle [69]. The incorporation of exogenous melatonin promotes the growth of tomato seedlings subjected to NaCl stress by elevating antioxidant enzyme activity, proline concentration, and glycine betaine levels, while concurrently diminishing glyoxalase activity, chlorophyll degradation, and ROS levels [82]. Moreover, preliminary melatonin administration to tomatoes in saline conditions results in an enhancement of Fv/Fm, biochemical cooling coefficient, and the proportion of Photosystem II center opening [70]. Melatonin plays an important role in enhancing resistance to salinity stress. The mechanism of melatonin involves photosynthetic pigments, enhancement of antioxidant defenses, and improvement of physiological and biochemical parameters, indicating the applicative potential of melatonin in the context of agriculture, especially under salinity stress conditions. Therefore, the use of melatonin can be an effective solution to address the challenges of plant productivity caused by salinity stress.

## 7. The Mechanism of Melatonin in Mitigating the Effects of Abiotic Stress

Plants are stationary organisms. Plants can solely modify their physical attributes to adapt to adverse environmental conditions. In response to a hostile environment, cells must undergo swift and remarkable transformations to ensure survival. A cell is delineated from its external surroundings by a plasma membrane. This membrane exhibits selective permeability to ions and other diminutive molecules. Melatonin is an amphipathic molecule that readily diffuses past the cell membrane into the cytoplasm and penetrates intracellular compartments. This molecule helps regulate stress response pathways by stabilizing membrane integrity, ion homeostasis, and enhancing the expression of defense genes to overcome unfavorable conditions.

Abiotic stress causes oxidative damage to plant cells through the accumulation of high levels of ROS. ROS are essential for growth and operate as secondary messengers in signal transduction [83]. However, high concentrations of ROS trigger cell damage [84]. The excessive accumulation of ROS has harmful effects, including electron leakage, lipid peroxidation in cell membranes, DNA damage, protein denaturation, carbohydrate oxidation, pigment degradation, and disruption of enzymatic activity [85]. Plants develop various strategies to regulate their growth and prevent cell damage under different environmental stress conditions [86]. Plants have effective protective mechanisms, including antioxidant systems, to maintain ROS balance and prevent its negative impact on cells. The development of plant adaptation strategies becomes very important to minimize cell damage and sustain survival under extreme environmental conditions.

**Figure 3:**
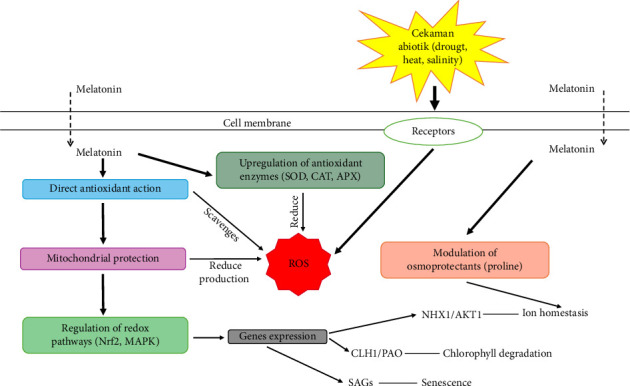
Melatonin mechanism in reducing oxidative stress.

Melatonin, as a new plant growth regulator, is suspected to play a role in responding to drought, temperature, and salinity stress, demonstrating its applicative potential in agriculture. The theory that melatonin supplementation reduces oxidative damage caused by stress by directly scavenging ROS and enhancing antioxidant and antioxidant enzyme activity has been confirmed in many experiments [87–90]. Several mechanisms of melatonin to reduce oxidative damage are presented in Figure 3. Melatonin in plants plays an important role in reducing oxidative stress caused by various abiotic stresses such as drought, extreme temperatures, and salinity. As an amphiphilic compound, melatonin can freely pass through cell membranes. Melatonin acts as a direct scavenger for free radicals, such as hydroxyl (OH-), superoxide (O_2_-), and hydrogen peroxide (H_2_O_2_), by neutralizing ROS before they can damage cells [54]. In addition, melatonin also enhances the activity of antioxidant enzymes such as superoxide dismutase (SOD), catalase (CAT), and ascorbate peroxidase (APX) [91], which effectively reduce ROS levels and protect cellular structures from oxidative damage. Thus, in addition to acting as a free radical scavenger, it also strengthens the plant's natural antioxidant defenses.

Melatonin is highly effective at scavenging ROS and preventing lipid peroxidation in biological membranes. Its strategic placement on the hydrophilic side of the lipid bilayer allows melatonin to directly neutralize these toxic reactants. At low concentrations, melatonin molecules align parallel to the lipid tails; however, at high concentrations, they arrange parallel to the bilayers, with one melatonin molecule connecting with two lipid molecules [92]. This ability of melatonin to counteract ROS and its unique molecular orientation within lipid membranes make it a crucial antioxidant in protecting cellular integrity from oxidative damage.

Melatonin plays a crucial role in regulating stomatal movement, particularly under drought stress. Drought stress often leads to stomatal closure to reduce water loss, but this can also limit CO_2_ uptake, negatively impacting photosynthesis [93]. Many studies have shown that melatonin influences the size of the stomatal aperture in plants. Microscopic examination of these structures showed a significant increase in the size of the stomatal aperture in the presence of melatonin [94–96]. Melatonin, a naturally occurring compound, can help plants manage this trade-off by promoting stomatal opening even under drought conditions. Melatonin promotes stomatal opening, which enhances CO_2_ uptake for photosynthesis, while also improving the plant's water use efficiency [97]. This dual effect helps plants tolerate drought by maintaining photosynthetic activity and preventing excessive water loss.

Melatonin can promote stomatal opening by regulating various physiological and molecular processes, such as the activity of ROS scavenging enzymes. While promoting opening, melatonin also helps plants optimize water use, preventing excessive transpiration and minimizing water loss [94]. Melatonin can influence the levels and signaling of ABA, a plant hormone that plays a key role in stomatal closure during drought [98]. By promoting stomatal opening, melatonin helps maintain or even increase photosynthetic activity under drought conditions.

Melatonin increases the expression of antioxidant genes and strengthens the plant's defense system. Melatonin activates redox signaling pathways through transcription factors such as Nuclear factor erythroid 2 (Nrf2) and the mitogen-activated protein kinase (MAPK) pathway [99]. Melatonin alters the expression of genes involved in stress such as Chlorophyllase 1 (CLH1), pheophorbide oxygenase (PAO), Sodium/hydrogen exchanger 1 (NHX1), and senescence-associated gene (SAG). The expression of these genes shows physiological responses in the form of oxidative damage such as chlorophyll degradation, ion homeostasis leakage, and plant aging [100]. This mechanism is supported by the protection of mitochondria and chloroplasts, organelles that play a crucial role in energy production and photosynthesis, which often become the main source of ROS during stress conditions [101]. By protecting these organelles, melatonin helps maintain redox balance and further reduce ROS production.

Melatonin has been shown to play an important role in protecting plants from excessive light damage through regulation of the xanthophyll cycle and nonphotochemical energy absorption (NPQ). Melatonin promotes the xanthophyll cycle and increases the size of the xanthophyll pool, which contributes to dissipating excess light energy [102]. Exogenous melatonin mitigated photoinhibition by accelerating the response rate of NPQ through the stimulation of violaxanthin de-epoxidase activity and enhancement of the de-epoxidation of xanthophyll [103, 104]. Xanthophyll cycle and NPQ are associated with stress responses [105]. Through this mechanism, melatonin shows its potential as a protective agent against light stress in plants, opening up opportunities for future agricultural applications.

Melatonin plays an important role in enhancing plant resistance to abiotic stress by regulating osmoprotectants, triggering the expression of stress tolerance genes, and protecting the structural integrity of cell membranes. Melatonin regulates the increase of osmoprotectants such as proline, which helps plants maintain cellular homeostasis when facing water stress [62]. In addition, melatonin also triggers the expression of stress tolerance genes that play a role in protecting protein structures and membrane functions. At the same time, melatonin reduces lipid peroxidation, protecting cell membranes from damage and maintaining their structural integrity [106]. Overall, melatonin plays a key role in maintaining cellular stability and enhancing plant resistance to abiotic stress. All these mechanisms contribute to the protection of plants against oxidative stress, support healthy growth, and maintain the vital functions of plants in challenging environmental conditions.

## 8. Conclusion and Future Research Directions

Tomato plants show various levels of natural melatonin content, ranging from 0.64 to 50.1 ng/g, with the highest amounts found in the leaves. Abiotic stress, such as drought and extreme temperatures, can significantly increase melatonin production, which plays an important role in plant development and adaptation to changing conditions. The biosynthesis of melatonin is induced by abiotic stress, hence augmenting plant tolerance. In tomatoes, the application of melatonin has been demonstrated to increase photosynthesis and enhance antioxidant activities in response to stress.

Exogenous melatonin can positively affect plant morphology and overall quality, especially during drought, temperature, and salinity stress. In addition, melatonin helps reduce oxidative stress by decreasing the accumulation of ROS and enhancing chlorophyll retention. Its ability to penetrate the cell membrane helps maintain cell integrity and ion homeostasis. As an important regulator in plant stress responses, melatonin is a promising candidate for agricultural strategies to enhance plant resilience to abiotic stress.

Some future research directions related to melatonin in plants include genetic and molecular studies to understand the biosynthesis mechanisms of melatonin and the pathways involved in plant responses to stress. Further research could explore the use of external melatonin under various stress conditions, such as drought, salinity, and extreme temperatures, to determine the optimal doses and effective application methods. Moreover, it is important to study the influence of environmental factors, such as light, temperature, and humidity, on the biosynthesis of melatonin in various plant species.

The investigation of the interaction between melatonin and other plant hormones, such as auxin, cytokinin, and ABA, can also provide insights into their cooperative roles in growth and stress response. The application of melatonin as an agent to improve crop yield and quality in sustainable agricultural systems, as well as its impact on plant resistance to pests and diseases, is also an important focus. In addition, the effects of melatonin on plant metabolite profiles can be investigated using a metabolomic approach, providing new insights into how melatonin affects plant physiology.

Lastly, the research on the role of melatonin in improving the resilience of plants to climate change is highly pertinent, with a particular focus on the capacity of plants to adjust to increasingly extreme conditions. This line of research is expected to improve the understanding of the function of melatonin and its applications in agriculture, as well as the potential for increasing plant resilience to various stresses.

## Figures and Tables

**Table 1:** Using database keywords.

Keywords	Scopus	ScienceDirect	Web of Science	PubMed	ProQuest	Total
Melatonin	52,942	52,397	49,123	33,962	36,756	225,180
Melatonin and plant	3356	10,579	2928	1877	12,657	31,397
Melatonin and plant and tomato	186	1934	249	118	3558	6045
Melatonin and plant and tomato and abiotic stress	53	1050	90	56	2612	3861

**Table 2:** Melatonin content (fresh weight) of tomato varieties.

Tomato variety	Melatonin (ng/g FW)	Reference
Bond	23.87 ± 2.02	[34]
Borsalina	8.2 ± 0.6	[34]
Catalina	4.1 ± 0.9	[34]
Lucinda	4.45 ± 0.05	[34]
Myriade	8.0 ± 1.3	[34]
Gordal	17.10 ± 1.21	[34]
Marbone	18.13 ± 2.24	[34]
Santonio	7.73 ± 1.22	[34]
Pitenza	14.2 ± 0.7	[34]
Platero	13.6 ± 2.5	[34]
RAF	50.1 ± 6.7	[34]
NKT 072	6.4	[35]
Isis	1.6	[35]
Jack	2.2	[35]
Ciliegia	0.64	[35]
Prico	3.7	[35]
Jesus	4.3	[35]
Optima	14.77	[35]
Micro-Tom	0.6–5.9	[36, 37]

**Table 3:** The melatonin content of the tomato organ.

Organ	Melatonin content (ng/g FW)	References
Leaf	142.5 ± 8.9	[39]
Leaf	4.6 ± 1.6	[38]
Stem	33.1 ± 2.6	[39]
Stem	5.3 ± 1.5	[38]
Root	10.2 ± 0.9	[39]
Root	3.1 ± 1.0	[38]
Flower	2.8 ± 1.2	[38]
Fruit	2.5 ± 0.5	[38]
Fruit	0.85 ± 0.05	[40]
Fruit	0.03 ± 0.002	[41]
Seed	39.4 ± 2.5	[38]

**Table 4:** The effect of melatonin on the morphology and physiology of tomato plants under drought, temperature, and salinity stress conditions.

Stress	Melatonin dosage (µM)	Application	Function	References
Drought	100	Sprayed	Improving seedling growth, root characteristics, and leaf photosynthesis	[61]
Drought	100	Sprayed	Improving the root system and reducing drought sensitivity	[62]
Drought	100	Sprayed	Increasing antioxidant activity, protecting the photosynthetic system from oxidative damage	[63]
Drought	50	Sprayed	Improving plant drought resistance by suppressing the expression of genes associated with linoleic acid catabolism	[64]
Heat	10	Sprayed	Increasing photosynthesis activity	[65]
Heat	50	Soaked	Effective in increasing lycopene accumulation and improving fruit quality	[66]
Heat	100	Sprayed	Improving heat tolerance in tomato seedlings by enhancing quality, antioxidant defense mechanisms, inducing the ascorbate–glutathione cycle, and biosynthetic pathways to reprogram polyamine (PA) and nitric oxide (NO) metabolism. Increasing the shoot and root weight of the plants	[58]
Heat	100	Sprayed	Increasing endogenous melatonin levels and photosynthetic pigments, enhancing CO_2_ assimilation. Improving the Fv/Fm ratio (maximum quantum yield of Photosystem II) and chlorophyll content, reducing reactive oxygen species (ROS) accumulation, decreasing the expression of respiratory burst oxidase (RBOHS)–related genes, and chlorophyll degradation	[67]
Heat	100	Sprayed	Lowering ROS values more than without melatonin, proving its potential as an antioxidant, reducing lower thylakoid membrane damage, increasing photosynthesis, and higher fruit yields	[63]
Heat	150	Doused	Modulating stomata/increasing stomatal conductance in tomato seedlings	[68]
Salinity	150	Sprayed	Reducing chlorophyll degradation and inhibiting Photosystem II (PSII) and oxygen-evolving complex (OEC) damage	[69]
Salinity	150	Doused	Controlling ROS levels and preventing damage caused by increased ROS under salinity stress	[70]
Salinity	100	Doused	Increasing the fresh weight and dry weight of tomato seedling shoots and roots, as well as increasing the chlorophyll content	[71]
Salinity	150	Doused	Improving plant growth and biomass by maintaining ionic homeostasis and reducing sodium absorption	[72]

## Data Availability

The data and any additional information will be available upon request from the corresponding author.
